# Editorial: Foods, dietary supplements, and herbal products treating the diseases of the 21st century: moving from traditional to scientific research

**DOI:** 10.3389/fnut.2024.1388181

**Published:** 2024-03-18

**Authors:** Ana Sanches Silva, Neha Garg, Shivraj Hariram Nile

**Affiliations:** ^1^Faculty of Pharmacy, University of Coimbra, Coimbra, Portugal; ^2^Animal Science Studies Centre (CECA), ICETA, University of Porto, Porto, Portugal; ^3^Associate Laboratory for Animal and Veterinary Sciences (AL4AnimalS), Lisbon, Portugal; ^4^Department of Medicinal Chemistry, Institute of Medical Sciences, Banaras Hindu University, Varanasi, Uttar Pradesh, India; ^5^Laboratory of Medicinal Plant Biotechnology, College of Pharmacy, Zhejiang Chinese Medical University, Hangzhou, Zhejiang, China

**Keywords:** dietary supplements, herbal extracts, foods, allergies, cancer, metabolic disease, SARS-CoV-2

The Research Topic of Frontiers in Nutrition entitled: “*Foods, dietary supplements, and herbal products treating the diseases of the 21st century: moving from traditional to scientific research*” contributed to explore the role of food, dietary supplements, and herbal products in the treatment of the diseases in a scientific mode, paving the road to bridge the gaps in their scientific acceptability.

This Research Topic includes total 10 papers, including four review papers, two systematic reviews, and four original research papers.

The review entitled “*From antiquity to contemporary times: how olive oil by-products and waste water can contribute to our health*” (Albini et al.) addressed the historical recognition of the benefits of olive oil and it is by products in a variety of fields, including cooking, skincare, and medicine. Olive mill waste water (OMWW), a byproduct of olive oil production, causes environmental issues while also providing a rich supply of phytochemicals with health advantages. OMWW extracts have been studied for their anti-angiogenic and chemopreventive properties, with prominent polyphenols including hydroxytyrosol (HT), verbascoside, and oleuperin. A particular extract, A009, displayed anti-angiogenic actions *in vitro* and *in vivo*, outperforming HT alone. A009 also showed possible cardioprotective effects by lowering chemotherapy-induced cardiotoxicity and pro-inflammatory markers in cardiomyocytes.

Several multinational research teams in the paper have tested extracts from OMWW and other olive byproducts for biological activity. According to this paper, the favorable results point to the potential of A009 as nutraceuticals, cosmeceuticals, or dietary supplements, notably in cancer prevention or co-treatment with anticancer conventional treatments. Furthermore, potential of OMWW extracts provide cardioprotective advantages offers up possibilities for use in cardio-oncology.

The review paper entitled “*Modulation of immune response by nanoparticle-based immunotherapy against food allergens*” (Krishna et al.) focuses on food allergies, specifically their treatment using nanoparticles in allergen-specific immunotherapy.

The increased global frequency of food allergies and their associated life-threatening anaphylactic events has resulted in limited treatment options, which primarily provide symptomatic relief. Recent advances in science and clinical practice aim to solve this issue by developing new treatments for allergic diseases. Despite improvements, current allergy immunotherapy has limitations in terms of long-term efficacy and safety, as evidenced by local side effects and the risk of anaphylactic reactions.

Ongoing research into the safety and efficacy of allergen immunotherapy has prompted the development of novel approaches such as intra-lymphatic immunotherapy. Furthermore, the use of nanoparticles in allergen immunotherapy is highlighted as a safer and more effective treatment. This manuscript describes a unique drug delivery approach that involves gradually administering specific allergens in increasing dosages in order to induce desensitization and tolerance. It stresses various administration routes, processes, and the use of nanoparticles in allergen-specific immunotherapy.

The research paper entitled “*Xanthophyll pigments dietary supplements administration and retinal health in the context of increasing life expectancy trend*” (Jurja et al.) addresses the effects of a sub-class of carotenoids, xanthophylls, in retinal health. These supplements are widely recommended for preventing retinal degenerative damage and slowing down the progression of age-related changes. Notably, these dietary supplements are recognized for their total antioxidant activity, as confirmed by the photochemiluminescence method using the Antioxidant Capacity in Lipid soluble-substances procedure. This study involved subjects with comparable ages and retinal age-related degenerative abnormalities, as well as a similar number of healthy individuals with normal retinas. Both groups of subjects were then administered similar dosages of xanthophyll pigments dietary supplements, with variations in the association of xanthophylls with vitamins and oligo-elements. After a three-year supplementation period, the subjects were reevaluated, and this paper emphasis the impact of these supplements on visual health.

The systematic review entitled: “*Impact of omega-3 fatty acids supplementation on the gene expression of peroxisome proliferator activated receptors-*γ, α *and fibroblast growth factor-21 serum levels in patients with various presentation of metabolic conditions: a GRADE assessed systematic review and dose–response meta-analysis of clinical trials*” (Ahmadi et al.) examined the impact of omega-3 fatty acid supplementation on the gene expression of peroxisome proliferator-activated receptors (PPAR-α and PPAR-γ) and serum fibroblast growth factor-21 (FGF-21) levels in people with various metabolic disorders. The analysis covered 15 trials found by a comprehensive search of various databases until April 2022.

Omega-3 fatty acids supplementation significantly increased PPAR-γ and PPAR-α gene expression compared to the control group. Overall, the results imply that omega-3 fatty acid supplementation may have a favorable effect on the regulation of adipose tissue-related genes in people with diverse metabolic disorders. However, more studies are needed to corroborate these findings and confirm the usefulness of this supplementation strategy in varied groups.

The systematic review entitled “*The metabolic effect of Momordica charantia cannot be determined based on the available clinical evidence: a systematic review and metaanalysis of randomized clinical trials*” (Laczkó-Zöld et al.) is a meta-analysis that assesses the efficacy of *M. charantia* L. (bitter melon) in treating metabolic syndrome, with a particular emphasis on its anti-diabetic properties. The study includes nine randomized controlled human trials with a total of 414 individuals and follow-up periods ranging from 4 to 16 weeks.

The meta-analysis, which followed the PRISMA statement, found no significant effects of bitter melon treatment above placebo in terms of change scores for most parameters. The bitter melon treatment had no significant influence on fasting blood glucose, HbA1c, HDL, LDL, total cholesterol, body weight, BMI, or systolic and diastolic blood pressure readings. The meta-analysis also found no significant changes in ALT, AST, or creatinine levels. The findings highlight the need for additional study, including properly conducted clinical studies with extended durations, to better understand the potential advantages and safety of *M. charantia* in treating metabolic syndrome.

The research paper entitled “*The impact of high-glucose or high-fat diets on the metabolomic profiling of mice*” (Xie et al.) aims to determine the effect of high-glucose and high-fat diets on metabolomic profiles in primary tissues of C57BL/6J mice. Mice were given either a high-glucose or high-fat diet for 8 weeks, and the levels of metabolites in their primary tissues were evaluated. This study highlights the strong impact of dietary composition on the metabolic profiles of primary tissues in mice, implying that metabolomics could be useful for detecting the development of sickness in animal models. When the metabolic profiles of the two diet groups were compared to those of a control group, the study found 32 metabolites in the high-glucose diet (HGD) group and 28 metabolites in the high-fat diet (HFD). The most significantly changed metabolites were amino acids (AAs). However, it is vital to recognize the limitations of this research, and there is still much need for further investigation in this area.

The research paper entitled “*Hypoglycemic effects of Dendrobium officinale leaves*” (Lv et al.) evaluated the hypoglycemic effects and processes of *D. officinale* leaves (EDL), with a focus on a portion of the plant that has received less attention than its stems. Male C57BL/6 mice were fed a conventional or high-fat diet, as well as normal or EDL-containing water, for 16 weeks. Mice fed a high-fat diet and treated with EDL showed considerably lower blood glucose levels and better glucose tolerance, whereas mice fed a low-fat diet showed no such effects. These findings shed light on the hypoglycemic potential of *D. officinale* leaves, helping to better understand the molecular pathways that enhance insulin sensitivity. The findings may help guide future research into isolating specific chemicals from EDL for the potential creation of hypoglycemic medicines, providing a theoretical underpinning for using *D. officinale* leaves in this setting.

The review paper “*Nutrients, herbal bioactive derivatives and commensal microbiota as tools to lower the risk of SARS-CoV-2 infection*” (Romani et al.) focuses on laying a solid scientific foundation and recommending complementary nutritional methods to aid in the prevention and treatment of SARS-CoV-2 infections. The authors explore the processes of viral entry, highlighting the possible significance of polyunsaturated fatty acids like α-linolenic acid and other micronutrients in suppressing SARS-CoV-2 and its entrance gateways. Herbal-derived pharmacological substances, certain microbial strains, and microbial-derived polypeptides are also addressed for their ability to prevent SARS-CoV-2 infection. Furthermore, the research thoroughly investigates the role of probiotics, micronutrients, and herbal-derived substances in boosting the immune response to SARS-CoV-2. By providing a comprehensive scientific backdrop, the authors hope to create a foundation for considering dietary tools as supplementary strategies in the battle against the current pandemic.

The review entitled “*Mechanism of the antidiabetic action of Nigella sativa and thymoquinone: a review*” (Shaukat et al.) addresses *Nigella sativa* (NS), a plant with a long history of traditional medicine. This review investigates the pharmacological and pharmacokinetic aspects of NS as herbal diabetic medicine, focusing on its effects on oxidative stress and Diabetes mellitus development. NS, notably its thymoquinone (TQ)-rich volatile oil, has received interest for its efficacy and safety in diabetic treatment. However, determining a precise therapeutic dose remains difficult. NS has been shown to reduce insulin resistance, enhance insulin signaling, decrease cyclooxygenase-2, upregulate insulin-like growth factor-1, and avoid endothelial damage in diabetes patients.

The research paper “*Characterizations of microRNAs involved in the molecular mechanisms underlying the therapeutic effects of noni (Morinda citrifolia L.) fruit juice on hyperuricemia in Mice*” (Liu et al.) addresses the therapeutic effects of noni (*Morinda citrifolia* L.) fruit juice on hyperuricemia and the underlying molecular pathways utilizing a potassium oxonate-induced mice model. Mice fed with noni fruit juice presented significantly lower serum uric acid (UA) and xanthine oxidase (XOD) levels. The study shows that noni fruit juice can treat hyperuricemia by decreasing XOD activity and lowering serum UA levels. Furthermore, the noni fruit juice group has considerably lower levels of serum creatinine and blood urea nitrogen than the model group, implying that noni fruit juice improves UA excretion without impairing renal function in mice. The study presented persuasive experimental evidence to warrant future investigation of noni fruit juice as a possible therapy for hyperuricemia.

Research contributions to this topic highlight the wide range of foods, dietary supplements, and herbal products that have shown promise in the treatment of twenty-first century diseases such as SARS-CoV-2, cancer, allergies, and metabolic disorders. These useful discoveries not only broaden our understanding of alternative treatment techniques, but also pave the path for future research and development of new solutions that take advantage of nature's healing capabilities. Embracing natural therapies may provide a holistic and complementary dimension to traditional medical tactics, creating a comprehensive and customized approach to disease management in the modern era.

## Author contributions

AS: Conceptualization, Formal analysis, Investigation, Supervision, Writing—original draft, Data curation, Funding acquisition, Methodology, Project administration. NG: Resources, Validation, Visualization, Writing—review & editing. SN: Validation, Visualization, Writing—review & editing.

